# Castleman disease complicated by rheumatoid arthritis and postoperative chylous leakage: A case report

**DOI:** 10.1097/MD.0000000000041559

**Published:** 2025-02-14

**Authors:** Wei Liu, Zhuoyan Tao, Rong Liang, Xinpeng Hu

**Affiliations:** a Department of Breast, Jinan University Affiliated Guangzhou Red Cross Hospital: Guangzhou Red Cross Hospital, Guangzhou, Guangdong, China; b Department of Breast Surgery, Shenzhen Futian District Maternal and Child Health Care Hospital, Shenzhen, Guangdong, China; c Department of Breast Surgery, The Affiliated Traditional Chinese Medicine Hospital of Guangzhou Medical University, Guangzhou, China.

**Keywords:** Castleman disease, chylous leakage, complications, rheumatoid arthritis

## Abstract

**Rationale:**

Castleman disease (CD) is a rare disorder characterized by nonspecific clinical presentations and imaging findings, making it prone to misdiagnosis and missed diagnosis. This report details the diagnosis and treatment process of a patient with CD complicated by rheumatoid arthritis (RA) who developed chylous leakage postoperatively, highlighting the potential risks of infection and nutritional depletion, aiming to enhance the understanding of CD.

**Patient concerns:**

A patient with CD complicated by RA developed chylous leakage postoperatively, which posed risks of infection and nutritional depletion.

**Diagnoses:**

The patient underwent a left axillary lymph node excisional biopsy on November 13, 2019, and was diagnosed with HV-CD based on pathology.

**Interventions:**

No further axillary lymph node dissection or adjuvant therapy was performed. Postoperatively, the patient developed significant chylous leakage from the biopsy incision, which resolved after 2 weeks of drainage, dietary modifications, and anti-infective treatment.

**Outcomes:**

Follow-up showed no disease progression, and the chylous leakage resolved with appropriate management.

**Lessons:**

A history of RA may be associated with the onset of CD. HV-CD generally has a favorable prognosis, and chylous leakage post-axillary lymph node biopsy, though rare, can be effectively managed with appropriate drainage, dietary control, and infection prevention.

## 1. Introduction

Castleman disease (CD), also known as angiofollicular lymph node hyperplasia or giant lymph node hyperplasia, is a rare disorder characterized primarily by lymphadenopathy. In 2018, it was included in the first list of rare diseases by the Chinese National Health Commission and 4 other departments. According to reports, CD commonly affects the mediastinum, with relatively few cases involving axillary lymph nodes.^[1]^ Chyle is a milky substance that enters the lymphatic system directly from the intestines, containing high levels of proteins, fats, and white blood cells. Chylous leakage is common after head, neck, or thoracoabdominal surgeries, but it is extremely rare after axillary surgery, with an incidence of < 0.5%.^[2]^ Reports of chylous leakage following axillary lymph node biopsy in CD patients are even rarer. However, the coexistence of CD with rheumatoid arthritis (RA) and postoperative chylous leakage remains rare. This case report aims to summarize clinical and therapeutic experiences from a case of CD complicated by RA and postoperative chylous leakage.

## 2. Case presentation

**Figure 1.**
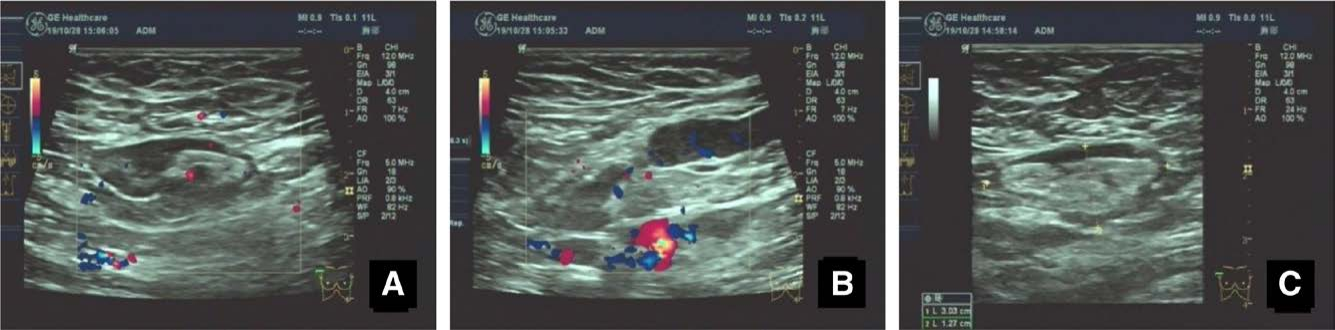
Doppler and ultrasound imaging of axillary lymph nodes. (A) Doppler blood flow image of the lymph nodes in the left axilla. (B) Doppler blood flow image of the lymph nodes in the right axilla. (C) Ultrasound image of the lymph nodes in the left axilla.

A 60-year-old female patient presented on November 6, 2019, following the detection of bilateral axillary lymphadenopathy during a routine health checkup 3 weeks prior. Ultrasound examination showed multiple enlarged lymph nodes in both axillae, some measuring up to 45 mm × 15 mm on the left (Fig. 1A) and 30 mm × 12 mm on the right (Fig. 1B). Color Doppler revealed rich blood flow signals in the left axilla (arterial spectrum Vmax: 17 cm/s, resistive index: 0.78) and gate-like flow in the right axilla. The ultrasound images confirmed bilateral axillary lymphadenopathy (Fig. 1C) and suggested the possibility of malignancy. The patient did not report palpable masses in the axillae and was asymptomatic for fever, night sweats, pain, or swelling.

The patient had a 6-year history of RA, well-managed with corticosteroids and immunosuppressants, and a 10-year history of hypertension, controlled with nimodipine. Her family history was significant for colon cancer in an elder sister, but no other familial patterns of disease were noted. Physical examination was unremarkable except for slight fullness in the left axilla, without palpable lymphadenopathy. No enlarged lymph nodes were detected in the right axilla or bilateral supraclavicular regions. Other superficial lymph nodes were also not enlarged.

Upon admission, laboratory investigations showed hemoglobin at 99.0 g/L and D-dimer at 1370 μg/L. Chest and abdominal compute tomography (CT) scans indicated multiple left axillary and left supraclavicular lymph nodes, and breast magnetic resonance imaging with contrast indicated, “Enlarged lymph nodes in the left axilla, possibly inflammatory. A lymph node measuring approximately 3.6 cm × 1.1 cm was observed, with visible hilum. No significantly enlarged lymph nodes in the right axilla. Multiple enlarged lymph nodes suggestive of lymphoma cannot be excluded,” revealed a 3.6 cm × 1.1 cm lymph node with a visible hilum in the left axilla, raising suspicion for lymphoma.

**Figure 2.**
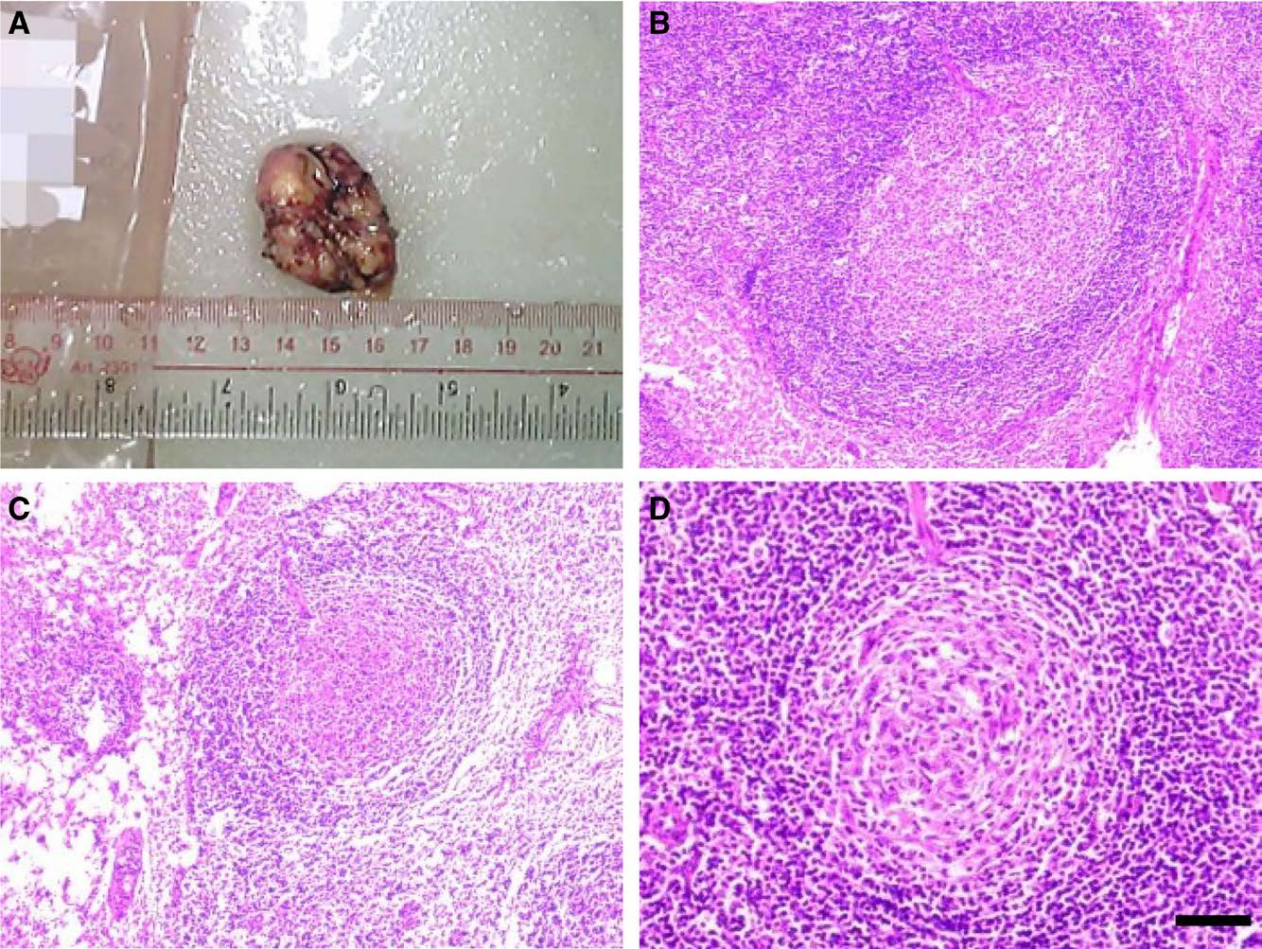
Gross and histopathological images of enlarged axillary lymph nodes. (A) Gross specimen of the enlarged lymph node in the left axilla. (B–D) Histopathological images under the microscope showing multiple enlarged lymph nodes from the left axilla, observed under 8 × magnification with a scale bar of 500 μm.

**Figure 3.**
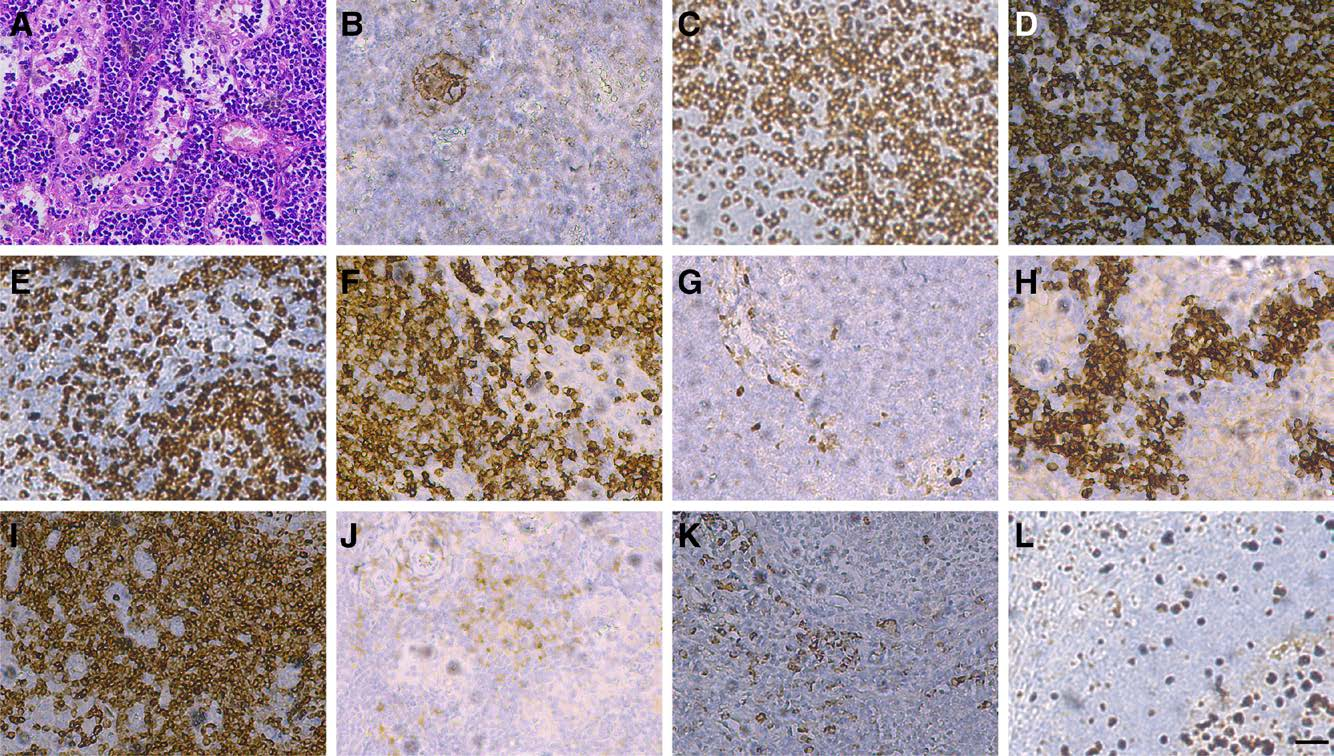
Histopathological and immunohistochemical analysis of the excised lymph node. (A) hematoxylin and eosin staining for pathological analysis. (B–L) Immunohistochemical staining for various markers: (B) CD21, (C) CD3, (D) CD20, (E) CD5, (F) CD43, (G) CyclinD1, (H) CD38, (I) Bcl-2, (J) kappa, (K) lambda, and (L) Ki67. All images were observed under 200 × magnification with a scale bar of 20 μm.

An excisional biopsy of the left axillary lymph node was performed on November 13, 2019, to establish a definitive diagnosis. Intraoperatively, a 4 cm × 3 cm × 1 cm gray-red, soft, encapsulated lymph node was completely excised (Fig. 2A). Histopathological examination (Figs. 2B–D, and 3A) demonstrated numerous enlarged lymphoid follicles with concentric (onion-skin) layering of lymphocytes and prominent vascular proliferation, consistent with hyaline vascular type CD. Immunohistochemistry showed CD21, CD3, CD20, CD5, CD43, CyclinD1(−), CD38, Bcl-2, Kappa (scattered+), Lambda (scattered+), Ki67 (about 10%), confirming the diagnosis (Fig. 3B–L).

The postoperative diagnosis was confirmed as hyaline vascular type Castleman disease (HV-CD). Three days postsurgery, following vigorous physical activity, the patient developed significant chylous leakage from the wound, producing up to 300 mL per day, without odor, redness, or fever. Laboratory analysis of the fluid showed a triglyceride level of 5.31 mmol/L, confirming the chylous nature of the leakage. The chylous leakage was managed with wound drainage, dressing changes, and oral antibiotics, resulting in a gradual reduction of wound secretion and complete healing within 2 weeks. The patient was advised to seek further oncological treatment but declined additional radiotherapy or chemotherapy.

In December 2019, PET-CT results indicated increased metabolic activity in the left axillary region (standardized uptake value ~5.8), likely postoperative changes, and mildly increased activity in bilateral axillary lymph nodes without evidence of disseminated disease. The patient remained asymptomatic and continued regular follow-ups. An ultrasound in October 2020 revealed stable nodules in both axillae with no significant progression. The patient has been asymptomatic with no significant disease progression to date.

## 3. Discussion and conclusions

CD is extremely rare, with an incidence of about 0.0025%.^[3]^ The pathogenesis of CD remains unclear. In the 1980s, interleukin-6 (IL-6) was identified as a potential key cytokine in CD pathogenesis.^[4,5]^ Subsequently, human herpesvirus-8 was detected in CD patients, suggesting that IL-6 and its homologs play crucial roles in the disease.^[6]^ IL-6 abnormalities are also implicated in anemia and RA. Studies show IL-6 correlates positively with hepcidin expression, leading to iron metabolism disturbances and anemia.^[7]^ The present case, like many others, also showed anemia, supporting this association. Additionally, IL-6 is highly expressed in RA patients, indicating a potential link between RA history and CD development.^[8,9]^ Given the central role of IL-6 in CD pathogenesis, anti-IL-6 therapies have become important treatment options. Tocilizumab and siltuximab, which target IL-6, have shown significant efficacy in reducing inflammation and controlling symptoms in CD patients.^[10,11]^ This aligns with the current case, where anemia and IL-6 abnormalities were observed, further supporting the association between IL-6 and CD. However, despite these findings, the link between IL-6 and CD remains insufficiently evidenced, and further studies are necessary to clarify its role.

CD is classified into hyaline vascular (HV-CD), plasma cell (PC-CD), and mixed types based on pathological features.^[12]^ HV-CD, the most common type, is characterized by increased lymphoid follicles and interfollicular vascular proliferation, accounting for about 90% of cases.^[13]^ CD is further classified into unicentric (UCD) and multicentric (MCD) based on the extent of lymph node involvement. MCD can be associated with immunocompromised states and human herpesvirus-8 infection.^[14]^ Clinically, UCD often presents as asymptomatic lymphadenopathy, while MCD exhibits systemic symptoms such as fever, night sweats, weight loss, anemia, and hepatosplenomegaly.^[15,16]^ Imaging findings are nonspecific, making pathologic biopsy the diagnostic gold standard.^[17]^ Histopathological examination, supported by immunohistochemical markers such as CD21, CD3, and Bcl-2, is crucial in differentiating CD subtypes and guiding treatment strategies.^[18]^ In the diagnostic process, we considered malignant lymphoma and other inflammatory diseases; however, the pathological and immunohistochemical findings confirmed HV-CD.

Effective treatments for CD are still under investigation. Surgical excision is considered the best treatment for UCD, often leading to clinical cure.^[19]^ MCD has a poorer prognosis and may require nonsurgical treatments like corticosteroids, cytotoxic drugs, and IL-6-targeted therapies, though results are not always satisfactory.^[20,21]^ Siltuximab, an anti-IL-6 monoclonal antibody, has shown significant efficacy in improving symptoms and quality of life in MCD patients, though some patients may not respond adequately.^[22,23]^ In this case, the patient underwent a biopsy for diagnostic confirmation, with PET-CT follow-up indicating residual disease but no further treatment pursued. The patient has remained stable for over 2 years. Given that the patient had unicentric HV-CD and remained stable, we opted against further treatment and chose regular follow-up observation instead.

Chylous leakage postaxillary surgery is rare, typically occurring within 1 to 4 days postoperatively.^[24]^ It may result from damage to abnormal branches of the thoracic duct draining the axilla.^[25]^ Diagnosis is often clinical, supported by typical chyle characteristics and elevated triglyceride levels in the leakage fluid.^[26]^ Current evidence and clinical experience suggest that low-to-moderate output chylous leakage can often be managed effectively with conservative measures. These include continuous wound drainage, dietary modifications to reduce chyle production (eg, a low-fat diet enriched with medium-chain triglycerides), and prophylaxis infection.^[27,28]^ Pharmacological therapies, such as somatostatin analogs, may be employed in cases of persistent leakage, while surgical intervention is generally reserved for high-output or refractory cases.^[29,30]^ In this patient, the chylous leakage was moderate, and no systemic complications, such as malnutrition or immune dysfunction, were observed. These factors supported the decision to adopt conservative management, which led to a successful resolution of the condition. Timely intervention with drainage and dietary adjustments effectively reduced leakage and prevented secondary complications.

The management of chylous leakage following axillary lymph node biopsy in CD patients, though rare, requires a multidisciplinary approach. Prompt identification and conservative management, including wound drainage, dietary modifications, and infection prevention, are essential to ensure optimal patient outcomes. The patient’s recovery without further complications and the absence of disease progression over 2 years highlight the effectiveness of this approach. However, long-term follow-up is crucial for monitoring potential recurrence or late complications of CD, particularly given the patient’s history of RA. Regular imaging and laboratory evaluations, including monitoring IL-6 levels and inflammatory markers, could help detect disease recurrence or progression early. Additionally, close monitoring of RA disease activity and ensuring continued control with immunosuppressive therapy are vital to reduce the risk of systemic complications that may exacerbate CD. A comprehensive follow-up plan combining clinical, laboratory, and imaging assessments should be tailored to the patient’s overall health status and disease history. The potential link between RA and CD via IL-6 suggests an avenue for further investigation. Understanding this relationship may improve diagnostic accuracy and lead to more targeted therapies. Future studies should focus on the molecular mechanisms underlying CD and its associations with autoimmune diseases to develop more effective management strategies. The complexity and challenges associated with diagnosing and managing CD, particularly when complicated by RA and postoperative chylous leakage, highlight the need for continued research and vigilant clinical management.

## Author contributions

**Data curation:** Wei Liu, Zhuoyan Tao, Rong Liang.

**Formal analysis:** Wei Liu, Zhuoyan Tao, Rong Liang, Xinpeng Hu.

**Funding acquisition:** Wei Liu.

**Writing – original draft:** Wei Liu, Xinpeng Hu.

**Writing – review & editing:** Wei Liu, Xinpeng Hu.
